# Effect of simulation-based emergency airway management education on the knowledge, skills and perceived confidence of medical interns

**DOI:** 10.1097/MS9.0000000000002376

**Published:** 2024-08-07

**Authors:** Samjhana Basnet, Sailesh P. Shrestha, Roshana Shrestha, Anmol P. Shrestha, Ashish Shrestha, Sandeep Sahu, Bhavana Mhatre, Prabhat Silwal

**Affiliations:** Deparments ofa General Practice and Emergency Medicine; b Anesthesia and Critical Care; c Dhulikhel Hospital, Kathmandu University School of Medical Sciences, Dhulikhel, Kavre, Nepal; d Deparment of Anesthesia, Sanjay Gandhi Post Graduate Medical Institute, Lucknow; e Department of Physiotherapy, PT School and Center, Seth GS Medical College and KEMH, Mumbai, India

**Keywords:** airway management, medical education, mixed methods study, simulation-based education

## Abstract

**Background:**

An effective airway management education program is a crucial part of the undergraduate medical education curriculum. Theoretical instructions and practical demonstrations are the major modalities of medical education in Nepal. Simulation-based education (SBE) programs have not yet been implemented effectively. The authors aimed to determine the effects of an SBE program on the knowledge, skills, and perceived confidence of medical interns regarding emergency airway management.

**Methods:**

This mixed methods study comprised both quantitative and qualitative components. The study participants were 47 medical interns who had participated in the SBE program.

**Results:**

The mean age of the 47 participants was 24.74 years. There were 33 (70.21%) male and 14 (29.79%) female participants. The knowledge, skills, and perceived confidence scores of the participants for airway management preparation, basic airway management, endotracheal intubation, and laryngeal mask airway (LMA) insertion improved significantly following the SBE program (*P*<0.001). Analysis of the participants’ feedback indicated that they largely approved of the SBE program. The majority of students and faculty expressed a willingness to include similar programs in the undergraduate medical education curriculum.

**Conclusion:**

This study demonstrated through quantitative and qualitative metrics that SBE can enhance the knowledge, skills, and perceived confidence in performing emergency airway management among medical interns. The authors recommend measures to include and effectively implement SBE in the undergraduate medical education curriculum of Nepal.

## Introduction

Highlights

- The knowledge, skills and perceived confidence scores of the participants for airway management preparation, basic airway management, endotracheal intubation, and laryngeal mask airway insertion improved significantly following the simulation-based education (SBE) program.
- Analysis of the participants’ feedback indicated that they largely approved of the SBE program.
- The majority of students and faculty expressed a willingness to include similar programs in the undergraduate medical education curriculum.
- We recommend measures to include and effectively implement SBE in the undergraduate medical education curriculum of Nepal.

Failure to appropriately manage a compromised airway contributes significantly to morbidity and mortality worldwide^1^. Poor education and lack of appropriate hands-on exposure to trainees are important causes of poor patient outcomes related to airway management^2^. Hence, it is crucial to include an effective airway management education program in the medical education curriculum.

The National Curriculum Framework for Undergraduate Health Professions Education in Nepal published by the Medical Education Commission in 2023 requires that medical students gain competencies in emergency airway management skills^3^. Traditionally, medical students in Nepal receive theoretical instruction and some practical demonstrations in airway management as a part of their clinical science education. However, in our experience, the knowledge and skills of medical students regarding airway maneuvers are often inconsistent and untested before entering clinical practice. Limited time and opportunities for hands-on practice further disadvantage students during their 1-year clinical internships to master their proficiency in airway management.

Medical universities worldwide are transitioning from classroom-based instruction to experiential teaching methods that simulate clinical settings^4,5^. Simulation-based education (SBE) has emerged as a safe and effective tool to allow medical students to hone clinical skills in a controlled and reproducible environment while also mitigating the ethical concerns of students practicing unfamiliar and acute care procedures on real patients^4–6^.

Through SBE, medical students are able to test their knowledge, receive structured feedback, and engage in experiential and reflective learning in a risk-free environment^6–8^. SBE has been shown to better prepare medical students and produce more confident and competent medical doctors^8–10^. The use of SBE is also associated with better patient outcomes^9^. Furthermore, SBE strengthens participants’ emotional, cognitive, and psychomotor skills, leading to positive changes in attitude and behavior in clinical practice^8,9^.

Implementation of SBE in the medical education system of Nepal has only just started. The National Curriculum Framework for Undergraduate Health Professions Education has recommended the use of SBE as a teaching method^3^. Furthermore, some instances of use of SBE for medical education in Nepal have been reported^11–14^. However, there are no published reports till date on the use of SBE in airway management education in Nepal. This study aims to determine the effects of an SBE program on the knowledge, skills, and perceived confidence of medical interns regarding emergency airway management. Our hypothesis is that SBE significantly improves knowledge, attitude and practice. We also evaluate the attitudes of the students and faculty towards the SBE program.

## Materials and methods

### Study design

This was a mixed methods study comprising both quantitative and qualitative components. A one-group pre-test-post-test quasi-experimental study represented the quantitative aspect. Qualitative component consisted of feedback from the interns and educators.

This mixed methods study follows an embedded design such that the primary emphasis is on the quantitative study and the qualitative approach serves to enhance the results^15^. Data collection and analysis were both done in a similar timeframe in a concurrent fashion^16^. Integration at the methods level was achieved through ‘connecting’ as the same sample population was used for both methods^16^. The contiguous approach to integration was adopted during the reporting of findings^16^.

### Setting

The study was conducted in the simulation center as well as the emergency department of a teaching hospital in Nepal. The SBE program was conducted in the simulation center over 3 days and 15–20 intern doctors participated on each day. Furthermore, simulation sessions consisting of 4–6 participants were conducted once every 2 weeks in the emergency department. The study was conducted from 1 December 2021 to 31 May 2022.

### Ethical considerations

Written informed consent was obtained from all the medical interns who participated in this study. Ethical approval was obtained from the Institutional Review Committee (IRC). The study conforms to the ethical guidelines of the Declaration of Helsinki as reflected in approval by the institution’s IRC. This study adhered to the STROCSS (Strengthening the Reporting of Cohort, Cross-Sectional and Case-Control Studies in Surgery) guidelines^17^.

### Participants

Out of 55 medical interns who were undergoing their mandatory rotating 1-year clinical internship at the teaching hospital, 47 attended the SBE program and were included in this study. The study participants had little to no prior experience in airway management.

### SBE program

All the trainers involved in the SBE program (*n*=5) are experts in airway management and are affiliated to the Department of General Practice and Emergency Medicine or the Department of Anesthesia of the teaching hospital. A workshop was conducted by a team of American Heart Association certified Advanced Cardiovascular Life Support (ACLS) course instructors to guide the trainers on the SBE program, simulation scenarios, simulation equipment, debriefing, and clinical skills assessment.

**Figure 1:**
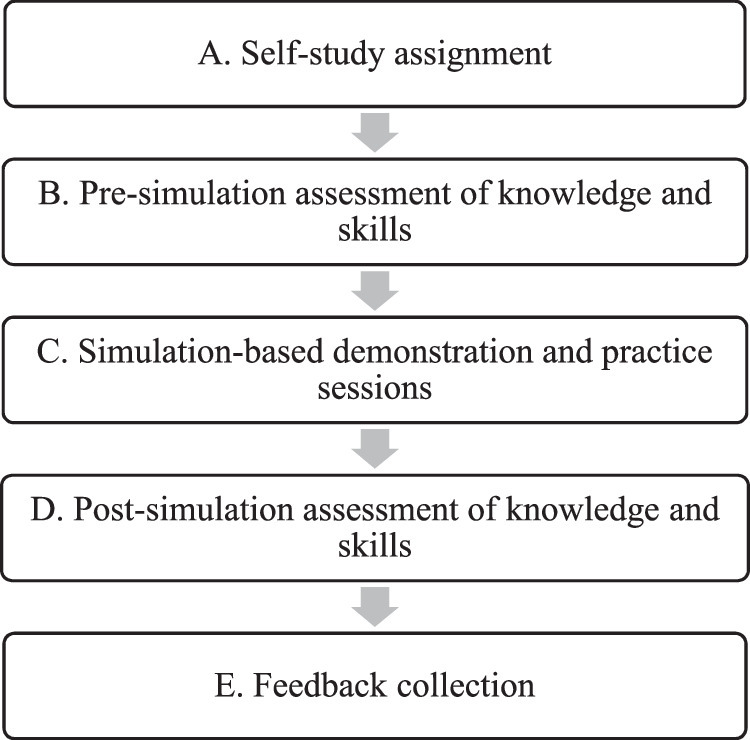
Workflow of simulation-based emergency airway management education.

The SBE program was conducted separately at the simulation center and the emergency department. Each participant underwent a half day simulation training at the simulation center followed by a simulation session at the emergency department. The method of SBE was the same for both these locations. Simulated clinical scenarios as well as the Laerdal and Ambu Airway Management Trainers, which are medium-fidelity manikins, were used. The workflow is described in Fig. 1.

**Figure 2:**
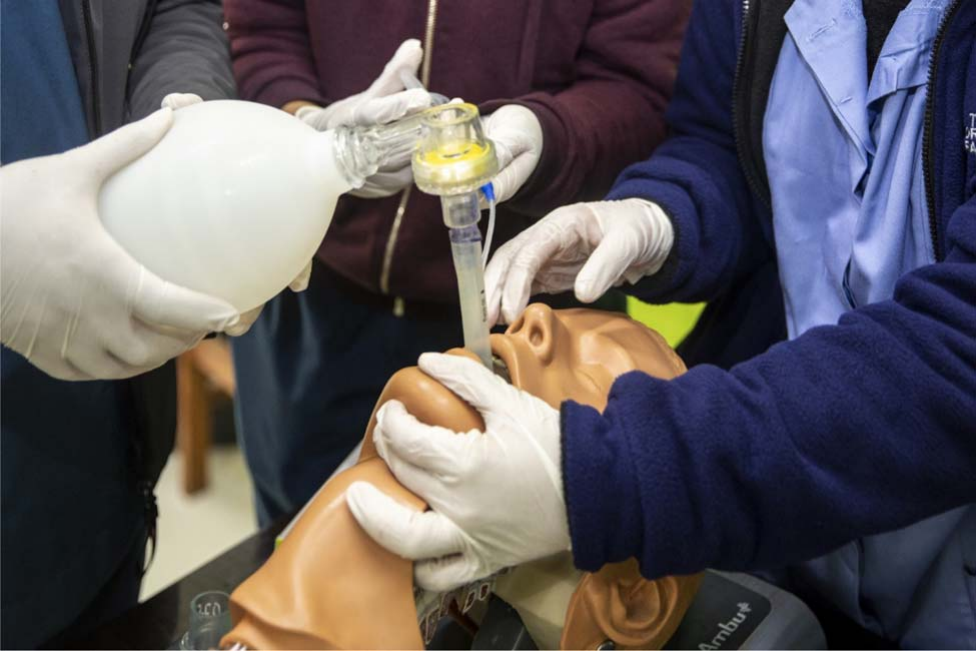
Equipment used to simulate. LMA insertion LMA, laryngeal mask airway.

**Figure 3:**
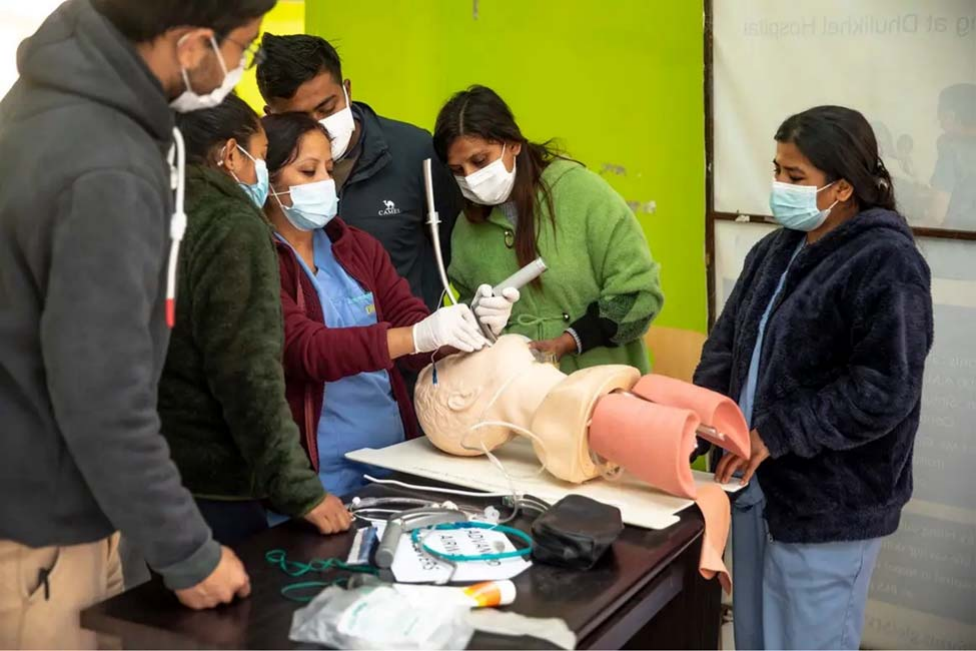
Simulation-based demonstration on endotracheal intubation.

- Self-study assignment
  All interns were asked to study assigned topics and review lecture materials pertaining to airway management before the day of the simulation exercises.
- Pre-simulation assessment of knowledge and skills (30 min)
  After arriving at their training sites, a two-part written exam was distributed to all interns. The first part of the assessment consisted of a survey of demographics, current level of medical training and self-reported confidence in performing airway maneuvers. The second part of the exam tested the interns’ clinical knowledge on airway management. For clinical skills assessment, students were scored on the basis of their performance in a simulated scenario requiring airway management. Assessment was done by the trainers who are experts in airway management.
- Simulation-based demonstration and practice sessions (four 1-h long sessions)
  Interns were grouped into teams of 4-6 members and each team underwent four simulation sessions at four different stations. Each session lasted for around an hour. The four stations were focused on (1) preparation for airway management, (2) basic airway management, (3) advanced airway management or endotracheal intubation, and (4) laryngeal mask airway (LMA) insertion. Each session consisted of pre-briefing, simulation and debriefing.
  Pre-briefing consisted of a brief introduction to the scenario and the simulation equipment. Some of the equipments used are shown in Fig. 2. Then, an interactive simulation-based demonstration relevant to the station was conducted by an expert in airway management as shown in Fig. 3. This was followed by simulation-based practice sessions where interns took turns leading the airway management scenario. After completion of the simulation, a debriefing session was conducted. The Pendleton method was used to structure these feedback sessions^18^. Emphasis was laid on first encouraging interns to reflect on what they felt went well and where they can improve during the simulation before offering positive affirmation of students’ strengths and recommendations on how interns can further enhance their performance.
  The simulation scenarios consisted of an unconscious and unresponsive patient (represented by the manikin) who required immediate airway intervention. In an ideal case, the interns would (1) prepare for airway management prior to the patient’s arrival to the emergency department, (2) perform an in-line immobilization, (3) perform basic airway maneuvers, then (4) attempt to perform endotracheal intubation which would fail by design, and finally (5) successfully insert a LMA. If airway management was appropriately performed, then the simulated patient would recover. However, if the interns had not appropriately completed the critical steps above, the patient would not survive.
- Post-simulation assessment of knowledge and skills (30 min)
  A second scenario was presented to the interns, which required airway management and the clinical skills of the interns were assessed. Finally, a second written examination was distributed to the interns. The first part of the exam was a survey where students were once again asked to rate their confidence in performing airway management. The second part of the exam consisted of the same questions that were included in the pre-simulation knowledge assessment.
- Feedback collection (20 min)

Expert faculty who participated in the simulation were asked to immediately submit their feedback via paper survey. Interns were asked to complete an online survey to collect feedback.

### Study variables and assessment tools

Information on the participants’ age, gender, previous airway management training, previous resuscitation training, and previous airway management experience were collected.

The simulation scenarios, knowledge assessment questionnaire and the skill assessment checklist were prepared based on the BLS and ACLS Provider Manual, 2020 by a team of experts in airway management who are also heart and stroke foundation Basic life support and American Heart Association certified ACLS course instructors.

Interns’ knowledge was assessed using a questionnaire consisting of 19 questions. Content validity of the questionnaire was verified by a panel of independent experts. After the development of the questionnaire, it was pilot tested on a batch of six interns who are not included in the final analysis. During the pilot testing, suggestions were collected from the faculty trainers and the interns and amendments were made to the questionnaire.

Clinical skills were assessed in four domains using structured scoresheets by the trainers, who are experts in airway management. Clinical skills pertaining to preparation for airway management, basic airway management, advanced airway management (endotracheal intubation) and LMA insertion were assessed. The scoresheet consisted of a total of 44 actions expected from the interns, each of which were scored from 1 to 5 (performance highly exceeded expectation- 5, performance exceeded expectation- 4, performance met expectation- 3, performance partially met expectation- 2, or performance did not meet expectation- 1). Content validity of the skills scoresheet was once again verified by a panel of independent experts. During the pilot study, the interns (*n*=6) were each evaluated by all the trainers (*n*=5). An inter-rater reliability analysis revealed that the intraclass correlation coefficient was 0.969 (95% CI 0.848–0.995, *P*<0.001), which demonstrates a high inter-rater reliability.

Interns’ perceived confidence was reported by the interns themselves on a scale of 1–5 (least confident- 1, strongly confident- 5).

The feedback forms consisted of open-ended questions asking the trainers’ and participants’ opinion on the SBE program. Furthermore, specific predetermined statements were also presented to the trainers and participants, to which they expressed their agreement or disagreement on a scale of 1–5 (1- strong disagreement, 2- disagreement, 3- neutral, 4- agreement, 5- strong agreement).

### Data analysis

Continuous variables were described in terms of mean, 95% CI, standard deviation (SD) and range whereas categorical variables were reported as frequencies and

percentages. Paired *t*-tests were used to assess the difference in the knowledge, skill and confidence scores before and after the SBE. A *P* value of less than 0.05 was considered statistically significant. All statistical analyses were performed using the Stata Statistical Software: Release 17. College Station, TX: StataCorp LLC. Statistical results should be interpreted with caution due to the small sample size and lack of correction for multiple testing.

For qualitative analysis, the answers to the open-ended questions were analyzed by four of the authors. The data were read, analyzed and summarized by them independent of each other. Then a discussion session was held among the four authors for identification and validation of the key themes included in the responses. Agreement or disagreement to specific statements were also analyzed and described.

## Results

A total of 47 medical interns participated in this study. The mean age of participants was 24.74 years. Among the participants, 33 (70.21%) were male and 14 (29.79%) were female. Sixteen (34.0%) interns had previously received training in airway management. Although 37 (78.7%) of the interns had previously witnessed airway management, only 5 (10.64%) had been directly involved in airway management in a clinical setting. The details are summarized in Table 1.

**Table 1:** Characteristics of the participants. (*n*=47)

Variables	*n* (%)
Age in years:	
Mean (standard deviation)	24.74 (0.84)
Range	22–27 (min–max)
Sex	Male: 33 (70.21)
	Female: 14 (29.79)
Previous airway management training	Received: 16 (34)
	Not received: 31 (66)
Previously witnessed airway management	Yes: 37 (78.7)
	No: 10 (21.3)
Previously involved in airway management	Yes, in a patient: 5 (10.64)
	Yes, in a manikin: 20 (42.55)
	No: 22 (46.81)

The pre-simulation as well as post-simulation mean scores of the interns in the domains of knowledge, skill and confidence are tabulated (Table 2). A Paired *t*-test analysis showed a significant improvement in knowledge, skills and perceived confidence scores after the SBE as portrayed in Table 2.

**Table 2:** Paired *t* test analysis of the difference in knowledge, skills and confidence assessed before and after the simulation sessions

Domain	Simulation location	Pre-test mean (standard deviation)	Post-test mean (standard deviation)	Mean difference	95% CI	*P*
Knowledge	*In situ* in the emergency department (*n*=47)	11.021 (2.18)	16.85 (1.73)	5.83	5.07–6.58	<0.001
	Simulation center (*n*=47)	13.66 (1.8)	17.17 (1.6)	3.51	2.86–4.15	<0.001
Skills	*In situ* in the emergency department: (*n*=47)					
	Preparation for airway management	10.48 (1.65)	21.61 (1.85)	11.12	10.40–11.84	<0.001
	Basic airway management	18.08 (2.61)	45.17 (3.5)	27.08	26.05–28.11	
	Advanced airway management	23.31 ( 2.23)	55.63 (3.57)	32.31	31.19–33.43	
	LMA	20.14 (0.36)	48.17 (3.49)	28.02	26.81–29.22	
	Simulation center: (*n*=47)					
	Preparation for airway management	16.70 (2.21)	23.19 (1.99)	6.48	5.54–7.43	<0.001
	Basic airway management	28.97 (7.62)	46.06 (3.39)	17.08	14.82–19.34	
	Advanced airway management	33.08 (6.21)	54.97 (4.30)	21.17	19.08–23.25	
	LMA	28.72 (6.42)	51 (4.02)	22.27	20.18–24.37	
Confidence	*In situ* in the emergency department (*n*=47)	1.44 (0.502)	4.08 (0.61)	2.63	2.41–2.86	*P*<0.001
	Simulation center (*n*=47)	3.59 (0.54)	4.46 (0.504)	0.87	0.66–1.08	*P*<0.001

LMA, laryngeal mask airway.

Out of the 47 participants, 43 voluntarily voiced their opinions about the SBE program. Their responses were analyzed. The following themes were predominant in the responses:

### Teaching method

Many participants liked the way the simulation was delivered as a “*real-life scenario*”, in a well-equipped setting. Comments included: “*the scenarios resembled real life and discussed how the cases should be managed”, “we were taught with a more practical approach”* “*video-assisted simulation and demonstration built confidence*”, “*interactive learning, and the practical brainstorming sessions increased my confidence*”, “*everything was well organized and well taught by the mentors*” and “received *practical knowledge and its real-time practical application*”.

### Learning environment

Some of the participants liked the learner-friendly and safe learning environment. One of the participants liked “*the specific attention given to an individual, equal opportunities to all, safe and reproducible learning environment*”. Similarly, one of the participants remarked “*the virtual world is becoming a platform for the management of real-life dreadful situations”*.

### Technical skills

Most of the participants liked that the SBE program helped them in “*skill development*” through “*interactive learning*”. The participants mentioned they were able to “*build confidence*”. Another participant mentioned “*simulation and practice guided by our trainer helped us identify every wrong technique and helped us rectify it every time*”.

### Non-technical skills

Most participants liked that the SBE program helped them in building *teamwork and situational awareness skills.* Some participants liked that it taught them decision-making skills. One participant highlighted that “*We were free to plan, decide, and act as a whole in a real-time manner on our ow*n” and another one said, “*we acted out a real case scenario and managed accordingly in a group”. Participants* also mentioned that it was “*a team-based and coordinated learning method”*.

### Identification of latent safety threats

One participant commented: *“We were given case scenarios and we would miss many things to do in the simulation and are prepared to not make those mistakes for what’s coming in real-life situations*”. Participants highlighted the “importanc*e of the preparation phase”*.

**Figure 4:**
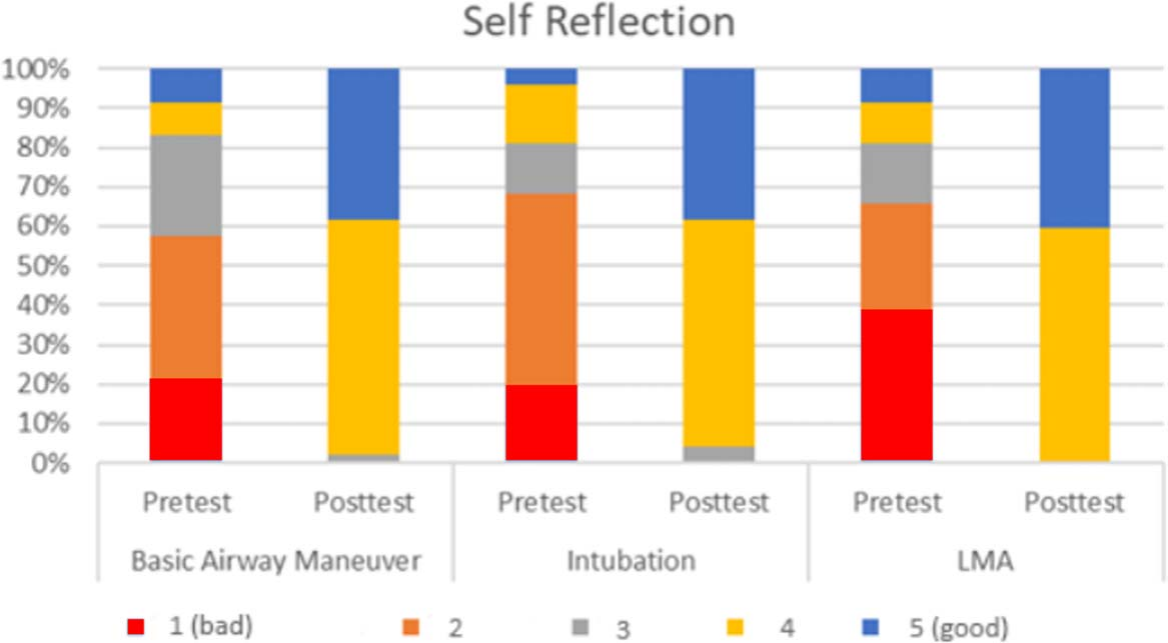
Interns’ self-evaluation of their technical skills. LMA, laryngeal mask airway.

Medical interns were asked to self-reflect on their technical skills before and after the simulation. They were asked to grade their own performance in the pre-test and post-test on a scale of 1–5 (1- bad, 5- good). The interns believed that their skills on basic airway maneuver, intubation and LMA improved after the simulation as shown in Fig. 4.

**Figure 5:**
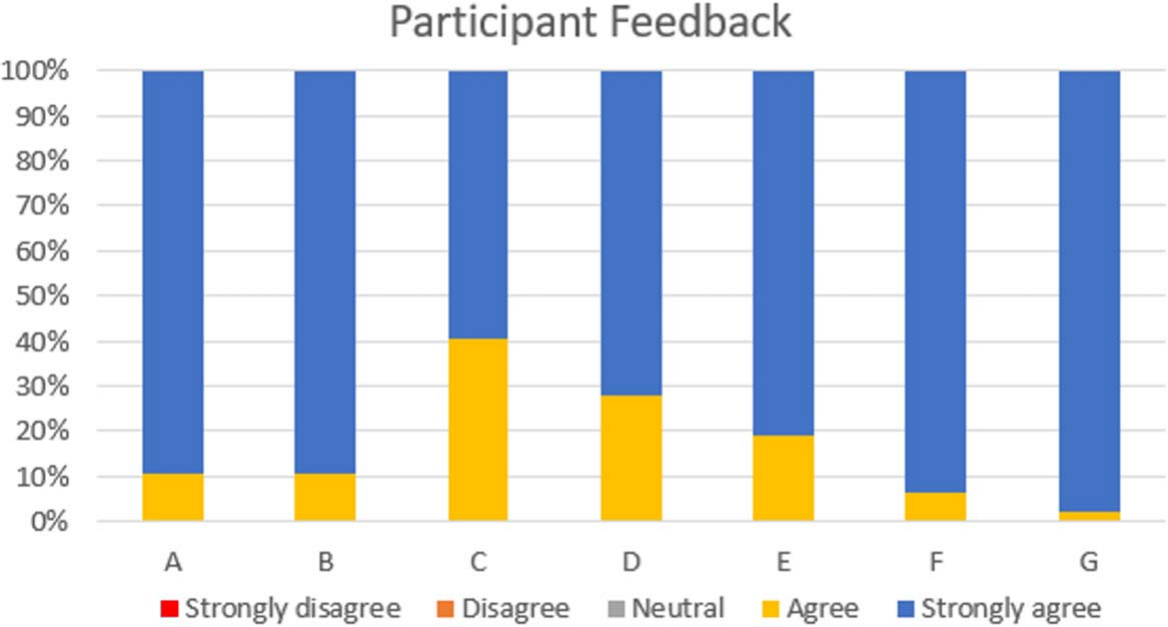
Participant interns’ feedback on simulation-based emergency airway management education program.

The interns also expressed their feedback in the form of agreement or disagreement to 7 predetermined statements on a scale of 1–5. The findings are described in Fig. 5.

**Figure 6:**
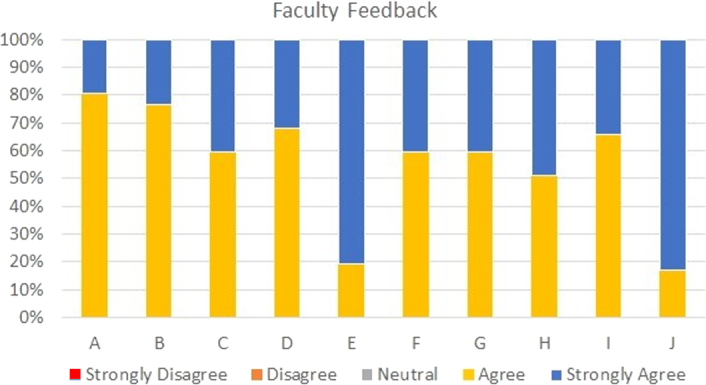
Faculty trainers’ feedback on the simulation-based emergency airway management education program.

Feedback was also collected from the faculty trainers in the form of agreement or disagreement to 10 predetermined statements on a scale of 1–5. The results are described in Fig. 6.

## Discussion

Airway compromise is considered a critical condition, and timely intervention to protect and maintain the airway is a major determinant of patient survival^19^. It is therefore imperative that medical students be thoroughly prepared to perform rapid and precise airway management before beginning their careers as medical doctors. The undergraduate medical curriculum of Nepal identifies airway management as an essential competency required for medical practice^3^.

The current medical school curriculum in Nepal is composed of two years of basic science education, two-and-a-half years of clinical science education, and one year of supervised clinical internship^20–22^. Students receive theoretical instruction and practical demonstration in airway management during clinical science education. However, there remains considerable variability in students’ knowledge and skills regarding airway management when they start their internships. The current curricular schedule affords little time for students to manually practice airway maneuvers in a clinical environment and raises legitimate ethical concerns about whether students are expected to practice unfamiliar procedures on critical patients.

SBE can bridge the gap between classroom-based education and clinical practice^6^. Implementing SBE would provide students with the space and resources to safely and repeatedly practice, and measurably improve, airway management skills that would otherwise be unreasonable or unfeasible for students to master on their current schedule. Existing literature identifies simulation-based learning as a safe and effective strategy for accelerating medical students’ mastery of airway interventions^9,23,24^. Additionally, simulation offers opportunities for risk-free practice and variations of clinical scenarios ensuring that students enter the real-life clinical environment ready for any patient encounter^25–27^.

Simulation training’s learner-centered approach to medical education has been shown to develop and reinforce students’ clinical knowledge as well as skills^9^. Our study demonstrated similar outcomes, when comparing pre- and post-simulation clinical knowledge and practical skill scores, students in this study exhibited significant improvement in knowledge and skill acquisition regarding preparation for airway management, basic airway maneuvers, endotracheal intubation, and LMA insertion.

It has been reported that SBE improves the cognitive skills and confidence of the participants of an airway management education program^9,28^. Our study reports similar findings. After completing the simulation, confidence scores significantly improved and the majority of students expressed feeling more self-assured in their clinical reasoning and technical abilities. Furthermore, faculty facilitators also observed that the simulation improved the teamwork, communication, and decision-making skills of the participants, all of which are essential elements of safe and efficient clinical care.

Feedback from the participants captured an overwhelmingly positive sentiment, with the majority of students commending the opportunity to iteratively practice new and/or complex airway techniques and constructively learn from their errors. Of note, the vast majority of students and faculty endorsed the future use of simulation training as a part of the curriculum. Research in resource-limited settings supports simulation-based medical training as a valuable and effective education tool^12,29^. However, there is a consistent emphasis placed on infrastructural context and local faculty involvement as key determinants of simulation program success^30,31^. The improvement in students’ performance together with the overwhelming agreement among students and faculty that our SBE program deserves broader application in the curriculum, suggests that our research team designed an SBE course appropriately tailored to the context.

This study has some limitations. The study was conducted on a limited sample of medical intern participants. While participants were able to perform airway maneuvers on the Laerdal and Ambu simulators, the manikins were unable to simulate certain physiological or anatomical factors that complicate airway procedures, such as bodily secretions or face/neck trauma. However, we believe the manikins were adequate and appropriate in our resource-limited context. We believe the instructor to student ratio in our SBE program was not optimal. A single faculty trainer was responsible for the guidance of up to five students during the simulation exercises. Due to resource limitations, we were unable to incorporate video recording of the simulations and video review during the debriefing sessions. In spite of the limitations, we believe this study is valuable in highlighting the role and need of SBE in the medical education system of Nepal and elsewhere.

## Conclusion

This study demonstrated through quantitative and qualitative metrics that SBE can enhance the knowledge, skills, and perceived confidence in performing emergency airway management among medical interns. This research adds to the growing body of literature supporting simulation training as a valuable tool in medical education.

We recommend the development, adaptation, and implementation of our emergency airway management simulation course in the undergraduate medical education curriculum of Nepal. Furthermore, the widespread integration and effective implementation of SBE programs into the undergraduate medical curriculum in Nepal is warranted.

## Ethical approval

Ethical approval was obtained from the Institutional Review Committee of Dhulikhel Hospital, Kathmandu University School of Medical Sciences (KUSMS-IRC), Kavre, Nepal on 05 December 2021, with approval number: 244/2021.

## Consent

Written informed consent was obtained from the patient for publication and any accompanying images. A copy of the written consent is available for review by the Editor-in-Chief of this journal on request.

## Source of funding

Not applicable.

## Author contribution

S.B., S.P.S., R.S., A.P.S., A.S., S.S., B.M., and P.S. contributed to the conceptualization, execution and conduction of the training program. S.B., S.P.S., R.S., A.P.S., A.S., S.S., B.M., and P.S. contributed to contributed to design, execution, writing the original draft, reviewing and editing of the manuscript. All the authors approved the final draft for publication.

## Conflicts of interest disclosure

The author declares no conflict of interest.

## Research registration unique identifying number (UIN)

Name of registry: Open Science Framework (OSF) registry

Link to Registration: https://doi.org/10.17605/OSF.IO/5AS7J

Unique identifying number: osf.io/5as7j

## Guarantor

Samjhana Basnet.

## Data availability statement

All data that support the findings of this study can be obtained by contacting the authors.

## Provenance and peer review

Not commissioned, externally peer-reviewed.
